# Protein Tyrosine Phosphatases PTP-1B, SHP-2, and PTEN Facilitate Rb/E2F-Associated Apoptotic Signaling

**DOI:** 10.1371/journal.pone.0097104

**Published:** 2014-05-08

**Authors:** Liza D. Morales, Edgar A. Casillas Pavón, Jun Wan Shin, Alexander Garcia, Mario Capetillo, Dae Joon Kim, Jonathan H. Lieman

**Affiliations:** 1 Edinburg Regional Academic Health Center, Medical Research Division, University of Texas Health Science Center at San Antonio, Edinburg, Texas, United States of America; 2 Department of Biology, University of Texas-Pan American, Edinburg, Texas, United States of America; 3 Department of Pharmacology, University of Texas Health Science Center at San Antonio, San Antonio, Texas, United States of America; 4 Department of Biology, South Texas College, McAllen, Texas, United States of America; Bascom Palmer Eye Institute, University of Miami School of Medicine; United States of America

## Abstract

To maintain tissue homeostasis, apoptosis is functionally linked to the cell cycle through the retinoblastoma (Rb)/E2F pathway. When the Rb tumor suppressor protein is functionally inactivated, E2F1 elicits an apoptotic response through both intrinsic (caspase-9 mediated) and extrinsic (caspase-8 mediated) apoptotic pathways in order to eliminate hyperproliferative cells. Rb/E2F-associated apoptosis has been demonstrated to be associated with the loss of constitutive transcriptional repression by Rb/E2F complexes and mediated by caspase-8. Protein tyrosine phosphatases (PTPs) PTP-1B and SHP-2 have been previously shown to be directly activated by loss of Rb/E2F repression during Rb/E2F-associated apoptosis. In this current study, we demonstrate that the PTEN tumor suppressor is also directly activated by loss of Rb/E2F repression. We also demonstrate that PTP-1B, SHP-2, and PTEN play a functional role in Rb/E2F-associated apoptosis. Knockdown of PTP1B, SHP2, or PTEN expression with small interfering RNA (siRNA) in apoptotic cells increases cell viability and rescues cells from the Rb/E2F-associated apoptotic response. Furthermore, rescue from apoptosis coincides with inhibition of caspase-8 and caspase-3 cleavage (activation). Our results indicate PTP-1B, SHP-2, and PTEN all play a functional role in Rb/E2F-associated apoptotic signal transduction and provide further evidence that PTP-1B, SHP-2, and PTEN can contribute to tumor suppression through an Rb/E2F-associated mechanism.

## Introduction

Apoptosis, or programmed cell death, is a tightly regulated cellular process that is required for the elimination of unessential, aberrant, or damaged cells. There are two types of apoptotic pathways: An extrinsic pathway which utilizes death receptors to trigger apoptosis and an intrinsic pathway which is mediated through a pathway involving the mitochondria [1]–[4]. A variety of external and internal stimuli and conditions can trigger these pathways leading to the activation of a family of cysteine proteases known as the caspases for the execution of cell death [3], [5]–[7]. The redundant duties of the apoptotic pathways ensure the maintenance of genomic integrity and tissue homeostasis which is vital to the prevention of cellular transformation and tumorigenesis. Nearly all cancers develop mutations to circumvent these apoptotic pathways [3], [6], [8].

Another hallmark of cancer is sustained cell proliferative signaling [8], [9]. Most tumors have genetic and epigenetic mutations which result in the loss of function of the retinoblastoma tumor suppressor protein, or Rb, which is a critical regulator of cell cycle progression at the transition from the G_1_ to S phase. Rb functions as a tumor suppressor and prevents cell cycle progression by binding to the E2F family of transcription factors to suppress transactivation of E2F-responsive genes that are required for entry into the S phase [4], [10], [11]. The Rb/E2F complex also actively represses gene transcription upon its occupation of target gene promoters and through its ability to promote histone modification; these events block promoter enhancers and prevent the transcription machinery from assembling [12]–[15]. In the event that Rb becomes mutated or functionally inactivated, E2F1 can elicit an apoptotic response to guard against aberrant cell proliferation [4], [10], [14], [16]–[21]. Until recent years, studies of the apoptotic mechanisms triggered by E2F1 had focused primarily on pathways initiated by E2F1 transactivation of pro-apoptotic genes. The most well-known of these pathways is the ARF/MDM2/p53 pathway: E2F transactivation of p19_ARF_ inhibits the E3 ubiquitin ligase MDM2. Inhibition of MDM2 allows p53 tumor suppressor to accumulate and activate pro-apoptotic genes, leading to the induction of an intrinsic apoptotic response [4], [18]. p53 is mutated or functionally inactivated in nearly all cancers [22], so p53-independent apoptotic signaling becomes equally essential to the elimination of aberrant or transformed cells.

Our previous work showed that loss of Rb/E2F constitutive transcriptional repression can initiate an apoptotic pathway that is mediated by caspase-8, (hereafter referred to as Rb/E2F-associated apoptosis). Rb/E2F associated apoptosis is characterized by inactivation of focal adhesion kinase (FAK) through dephosphorylation by protein tyrosine phosphatase (PTP), which triggers a robust p53-independent apoptotic response [23]. It has been established that FAK is overexpressed in a wide-range of tumors and it plays a critical role in the mechanisms contributing to tumor cell proliferation, survival, and migration [24]–[31]. For this reason, it has been recognized as a significant target for anti-cancer therapies [24]–[30]. We previously demonstrated that loss of Rb/E2F repression led to the transcriptional expression of two FAK-associated phosphatases: PTP-1B (protein tyrosine phosphatase 1B, encoded by *PTPN1*) and SHP-2 (SH2 domain-containing tyrosine phosphatase-2, encoded by *PTPN11*) [23], [32]. Another PTP that has been implicated in FAK regulation is PTEN (phosphatase and tensin homologue deleted on chromosome 10, encoded by *PTEN*) [33]–[35]. PTEN is a dual specificity phosphatase that is involved in a variety of cellular processes including cell cycle progression, cell proliferation, the DNA damage response and apoptosis. PTEN directly dephosphorylates FAK at the Tyr^397^ residue, its key autophosphorylation site, to negatively regulate integrin-mediated signaling [33], [35], [36]. More interestingly, PTEN is a confirmed tumor suppressor. *PTEN* is the second most mutated or deleted gene in human cancer after the critical tumor suppressor gene *p53*; these mutations most often arise in glioblastomas, malignant melanomas, endometrial cancer, and prostate cancer [33], [37]–[42]. In this work, we show that, like PTP-1B and SHP-2, E2F1 specifically regulates the *PTEN* gene. We also show that expression of PTP-1B, SHP-2, and PTEN facilitates Rb/E2F-associated apoptosis. Collectively, these studies indicate that these three PTPs can contribute to the Rb/E2F-associated apoptotic response and further provide details of a novel mechanism by which the PTPs PTP-1B, SHP-2, and PTEN can play a role in tumor suppression.

## Materials and Methods

### Cell Culture

EH1 cells were derived from a U2OS osteosarcoma cell line and modified to conditionally express an estrogen receptor (ER)-fused dominant negative E2F1 (dnE2F1) protein as previously described [23], [32], [43]. The ER-dnE2F1 cells (gift of D. Dean) were maintained in Dulbecco’s modified Eagle’s medium with 10% fetal bovine serum, 400 µg/ml zeocin, 150 µg/ml hygromycin B and 300 µg/ml G418 [23], [43]. Treatment with 1-thio-β-D-galactopyranoside (IPTG) (1 mM, Sigma-Aldrich Co., Saint Louis, MO) resulted in activation of p16Ink4a to establish endogenous Rb-mediated G1 arrest, and treatment with 4-hydroxytamoxifen (4-OHT or tamoxifen) (100 nM, Sigma-Aldrich Co.) resulted in activation and nuclear localization of dnE2F1 [23], [32], [43].

### Flow Cytometry

ER-dnE2F1 cells (approximately 0.5×10^5^ cells per ml) were activated (induced) by addition of IPTG and 4-OHT to trigger apoptosis. Control ER-dnE2F1 cells (uninduced) at time 0 h were treated with IPTG only. At the indicated time points, floating and adherent cells were collected and fixed in ice-cold 75% ethanol for at least 48 h, stained with PI/RNase staining buffer (BD Pharmingen, San Jose, CA), and analyzed for DNA content on a FACSAria II Cell Sorter (BD Biosciences, San Jose, CA). For experiments with RNA interference, ER-dnE2F1 cells were transfected with 4 nM of siRNA specific for PTPN1, PTPN11, PTPN12, PTEN, or scrambled control using RNAi Max (Invitrogen, Carlsbad, CA) 48 h prior to treatment with IPTG and 4-OHT. Apoptosis was induced for 48 h and then cells were fixed, stained, and analyzed as described. Uninduced cells transfected with scrambled siRNA were used as control. Data analysis was performed with FlowJo software. Percent rescue from apoptosis was calculated as (1– (% subG1_Experimental_/% subG1_Induced_))×100. Cells were imaged under bright field using a Leica DMI 6000 microscope using a 10X ocular lens and a 10X objective lens. For reproducibility and comparison purposes, microscope settings were kept identical among all samples. Three random images of the field of view were captured for each dish of cells and the number of cells in each image was manually counted using ImageJ software. Data are reported as mean + standard deviation of the mean. Statistical analysis of the significant difference (*p*<0.05) between induced cells transfected with scrambled siRNA and induced cells transfected with PTP-1B, SHP-2, or PTEN siRNA was determined by T-test for Equality of Means.

### Quantitative Real-time Polymerase Chain Reaction (qPCR)

Total RNA was collected and isolated from cell cultures treated with IPTG (uninduced) or IPTG and 4-OHT (induced) at 0 h, 1.5 h, 6 h, 12 h, 24 h, and 48 h using RNeasy® kit (Qiagen, Valencia, CA). The concentrations of the RNA samples were determined using the NanoDrop ND-1000 spectrophotometer. cDNA was generated with 0.5 ng of RNA using Superscript® III Reverse Transcriptase (Invitrogen) with the RETROscript® kit (Ambion, Grand Island, NY). Amplification was carried out in the 7900HT Fast Real-Time PCR System (Applied Biosystems, Grand Island, NY) using Taqman® Gene Expression Assay (Applied Biosystems) FAM-labeled probes for human gene *PTEN* and human GAPD (GAPDH) Endogenous Control (FAM/MGB probe) according to manufacturer’s instructions. The threshold C_T_ values were generated by the Applied Biosystems RQ Manager software version 1.2, and the experimental ΔC_T_ values were normalized to the ΔC_T_ values of the GAPDH endogenous control. Relative expression was calculated using the comparative C_T_ method (2^−ΔΔCT^). Experiments were repeated in triplicate and data are reported as mean + standard deviation of the mean. Statistical analysis of the significant difference (*p*<0.05) between uninduced and induced mRNA expression levels was determined by T-test for Equality of Means (Data not shown).

### Chromatin Immunoprecipitation (ChIP)

The TRANSFAC® Professional Software (http://www.biobase-international.com/) was used to identify putative E2F binding sites in the human *PTEN* gene promoter, and the sequence ccGCGAAc was identified within –3337…–3068 of the promoter region. Sequence-specific primers for *PTEN* were generated using DNASTAR® Lasergene Version 8 Sequence Builder software: PTEN forward, 5′–ATGGTAGCAGTGCAGCTGATGTG and PTEN reverse 3′–GCCTGACATTAAGAGTGACTT. ChIP assays were performed using an E2F1 specific antibody (Anti E2F1 clones KH20 and KH95, Millipore, Billerica, MA) or negative control mouse IgG antibody (Santa Cruz Biotechnology, Santa Cruz, CA) with the ChIP-IT Express Kit (Active Motif, Carlsbad, CA) according to manufacturer’s instructions. Briefly, induced cells from one 15 cm plate were fixed with 37% formaldehyde for 8 min. Cells were dounced on ice, and the nuclear lysates were sonicated 10×20s at 100% power using a sonicator to shear chromatin to an average size of 800 bp. IP’s were carried out with 100 µl of the sheared chromatin and 3 µg of antibody overnight at 4°C. Nuclease-free water was substituted for chromatin or antibody during parallel IPs as additional negative controls. PCR amplification of human *PTEN* and mouse *GAPDH* (control) was performed with 0.2 µl chromatin per reaction using GoTaq® Green Master Mix (Promega, Madison, WI) according to manufacturer’s instructions.

### Western Blot Analysis

Total protein lysates were prepared as follows: cells were lysed in ice-cold RIPA lysis buffer (50 mM Tris pH 8.0, 150 mM NaCl, 1.0% IGEPAL CA-630, 0.5% Sodium Deoxycholate, 0.1% SDS) containing 1∶100 protease inhibitor and phosphatase inhibitor cocktail 2 and 3 (Sigma-Aldrich Co.). Clear lysates were obtained by centrifugation at 4°C for 10 min at 13,000 rpm in a refrigerated microcentrifuge. Protein concentrations were determined using the BCA assay (Pierce, Rockford, IL) according to the manufacturer's instructions. Equal amounts (15–30 µg) of the protein samples were resolved using SDS-PAGE. The samples were then transferred onto a nitrocellulose membrane using an electroblotting method. The membrane was incubated overnight at 4°C with primary antibody, followed by incubation with a horseradish peroxidase-conjugated secondary antibody. Chemiluminescent detection reagents (GE Healthcare Bio-Sciences, Pittsburgh, PA) were used to detect immune-reactive protein. The following antibodies were used: anti-PTP-1B; anti-SHP-2; anti-PTEN (D4.3) XP; anti-caspase-3; anti-cleaved caspase-3 (Cell Signaling Technology Inc., Beverly, MA); (Santa Cruz Biotechnology) and anti-β-actin (Sigma-Aldrich Co.). The integrated optical density (IOD) measurements were taken using GelPro 3.1 software. The experimental IOD values were normalized to the IOD values of the β-actin loading control. Relative expression was calculated as IOD_Experimental_/IOD_Control_×100%.

### Cell Viability Assays

ER-dnE2F1 cells were plated (approximately 0.5×10^5^ cells per ml) onto a 6-well dish in triplicate and incubated overnight at 37°C and 5% CO_2_. Cells were transfected with 4 nM of siRNA specific for PTP-1B, SHP-2, or PTEN or scrambled control using RNAi Max (Invitrogen) for 48 h, and then cells were treated with IPTG and 4-OHT for 48 h. Cell viability was assessed by a formazantetrazolium salt (WST) assay with the Cell Counting Kit-8 (CCK8) (Dojindo, Rockville, MD). Uninduced cells transfected with scrambled siRNA were used as control and “medium only” was used as a control for background. Briefly, conditioned medium was replaced with fresh medium and 1∶100 CCK8 reagent was added to each well. Cells were incubated at 37°C for 30 min and absorbance was quantified at 450 nm on a spectrophotometer plate reader. Cell viability was calculated as ((OD_Experimental_−OD_Background_)/(OD_Control_−OD_Background_))×100%. Experiments were repeated in triplicate and data are reported as mean + standard deviation of the mean. Statistical analysis of the significant difference (*p*<0.05) between induced transfected with control siRNA and induced transfected with PTP-1BsiRNA, SHP-2 siRNA, or PTEN siRNA was determined by T-test for Equality of Means. Cell viability was also assessed for uninduced cells transfected with siRNA and “medium only” containing siRNA (Data not shown).

### Caspase Activity Assay

Caspase activity was measured by a colorimetric assay kit (Biovision, Milpitas, CA) using 100 µg of total protein lysate according to the manufacturer’s instructions. Briefly, ER-dnE2F1 cells were transfected with gene specific siRNA or scrambled control prior to treatment with IPTG and 4-OHT as mentioned above. Apoptosis was induced for either 24 or 48 h and then floating and adherent cells were pelleted. Uninduced cells transfected with scrambled siRNA were used as control. Cells were resuspended and lysed in chilled cell lysis buffer. Clear lysates were incubated with a labeled substrate specific to caspase-3 or caspase-8 in a 37°C water bath for 2 h and then light emission from cleavage of the labeled substrate was quantified at 405 nm on a spectrophotometer plate reader. Fold increase was calculated as (OD_Experimental_–OD_Buffer_)/(OD_Uninduced_–OD_Buffer_). Experiments were repeated in triplicate and data are reported as mean + standard error of the mean. Statistical analysis of the significant difference (*p*<0.05) between experimental and induced (control) data was determined by T-test for Equality of Means.

## Results

### *PTEN* is a Direct Target of Rb/E2F Constitutive Transcriptional Repression

Because PTEN is a major tumor suppressor and it is able to regulate FAK [33]–[35], [37]–[41], we wanted to investigate the potential role of PTEN in the Rb/E2F-associated apoptotic pathway, a signaling pathway mediated by FAK dephosphorylation [23]. To study this pathway we utilized an osteosarcoma cell line that conditionally expresses a dominant-negative mutant of E2F1 which localizes to the nucleus upon activation with tamoxifen (ER-dnE2F1). The dnE2F1 lacks the E2F transactivation domain that contains the Rb binding site. Overexpression of the dnE2F1 leads to the displacement of Rb/E2F complexes bound to the E2F binding site(s) within the promoters of E2F-responsive genes, including pro-apoptotic genes [23], [43]. Displacement of Rb/E2F by dnE2F1 simulates loss of Rb/E2F constitutive repression and triggers Rb/E2F-associated apoptosis, which is characterized by the accumulation of cells with a<2N DNA content [23], [32]. Consistent with previous studies, ER-dnE2F1 cells induced with tamoxifen to undergo Rb/E2F-associated apoptosis exhibited a rapid apoptotic response with an increasing percentage of cells undergoing cell death over a 48 h period (Data not shown).

**Figure 1:**
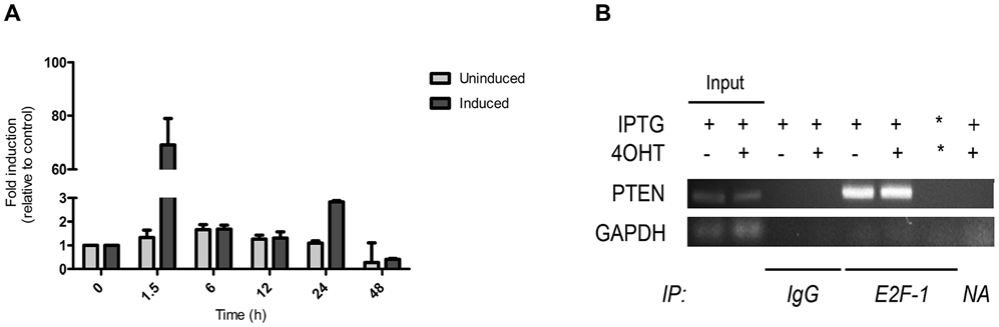
*PTEN* transcription is directly regulated by Rb/E2F complexes. ER-dnE2F1 cells were treated IPTG and with 4-OHT to induce Rb/E2F-associated apoptosis. Uninduced control cells were left untreated. (A) Total RNA was isolated at the indicated time points following induction of apoptosis and used for qPCR analysis for two independent experiments performed in triplicate. Results are expressed as the mean + standard deviation. (B) Chromatin was isolated from the cells, sheared and immunoprecipitated with control mouse IgG or E2F1 antibody. A ∼300 bp sequence within the PTEN promoter region was amplified by PCR. GAPDH was used as control. (−) Untreated; (+) Treated; (NA) No antibody negative control; (*) No chromatin negative control.

Promoter analysis of the *PTEN* tumor suppressor gene identified several putative binding sites for E2F (Data not shown) which suggested it may be regulated by Rb/E2F complexes and thereby it could be de-repressed by induction of dnE2F1 expression during Rb/E2F-associated apoptosis. To determine whether or not *PTEN* transcription is activated by this apoptotic response, quantitative real-time PCR (qPCR) was performed on uninduced ER-dnE2F1 cells (control) and induced ER-dnE2F1 cells over a 48 h time period (Fig. 1A). *PTEN* mRNA expression in induced cells peaked at 1.5 h after initiation of Rb/E2F-associated apoptosis by 69-fold relative to uninduced cells. PTEN mRNA expression remained unchanged in uninduced cells, which suggests that *PTEN* transcription is activated by loss of Rb/E2F repression. The house-keeping gene glyceraldehydes-3-phosphate dehydrogenase (*GAPDH*) was used as an internal control for qPCR. To verify that *PTEN* transcription is directly regulated by Rb/E2F complexes, Chromatin Immunopreciptiation assays (ChIP) were performed using sequence-specific probes for a putative E2F1 binding site identified within the *PTEN* promoter (Fig. 1B). Probes for the house-keeping gene *GAPDH* were used as control. The E2F1 antibody used for the assays targets the N-terminus of the E2F1 protein; therefore it is able to detect both exogenous dnE2F1 and endogenous E2F1 in complex with Rb [23], [32]. The results clearly showed that both IPTG-activated Rb/E2F complexes (in uninduced cells) and 4-OHT-activated dnE2F1 (in induced cells) specifically bound to the *PTEN* promoter so that the expected 300 bp PCR product was produced following chromatin immunoprecipitation with E2F1 antibody in comparison to the negative controls.

**Figure 2:**
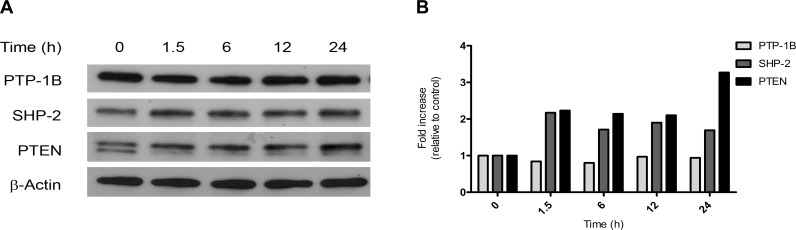
SHP-2 and PTEN protein expression increases during Rb/E2F-associated apoptosis. ER-dnE2F1 cells were treated with 4-OHT for the indicated times to induce Rb/E2F-associated apoptosis. Control cells (0 h) were left untreated. (A) Total protein was isolated from lysated cells at the indicated time points following induction of apoptosis, resolved by SDS-PAGE, and immunoblotted with antibodies specific for PTP-1B, SHP-2, PTEN and β-actin. (B) Relative expression levels of PTP-1B, SHP-2, and PTEN were quantified by densitometry. (h) Hours.

Together, the data confirms that the Rb/E2F complex directly suppresses *PTEN* transcriptional expression. Therefore, under conditions where Rb is functionally inactivated, the loss of Rb/E2F constitutive repression would allow for the up-regulation of *PTEN* and the subsequent expression of the protein. Western blot analysis of protein lysates collected from ER-dnE2F1 cells at 0, 1.5, 6, 12, and 24 h after induction of Rb/E2F-associated apoptosis exhibited an increase in SHP-2 and PTEN protein expression relative to the uninduced control (0 h), which is consistent with the transcriptional data (Fig. 2A). SHP-2 and PTEN protein expression levels increased 2-fold 1.5 h after induction of apoptosis and remained elevated for up to 24 h (Fig. 2B). PTP-1B protein expression levels remained relatively unchanged. The results imply that PTEN along with the previously identified PTPs, PTP-1B and SHP-2, may play an important physiological role in Rb/E2F-associated apoptosis.

### Loss of PTP Expression during Rb/E2F-associated Apoptosis Recovers Cell Viability

**Figure 3:**
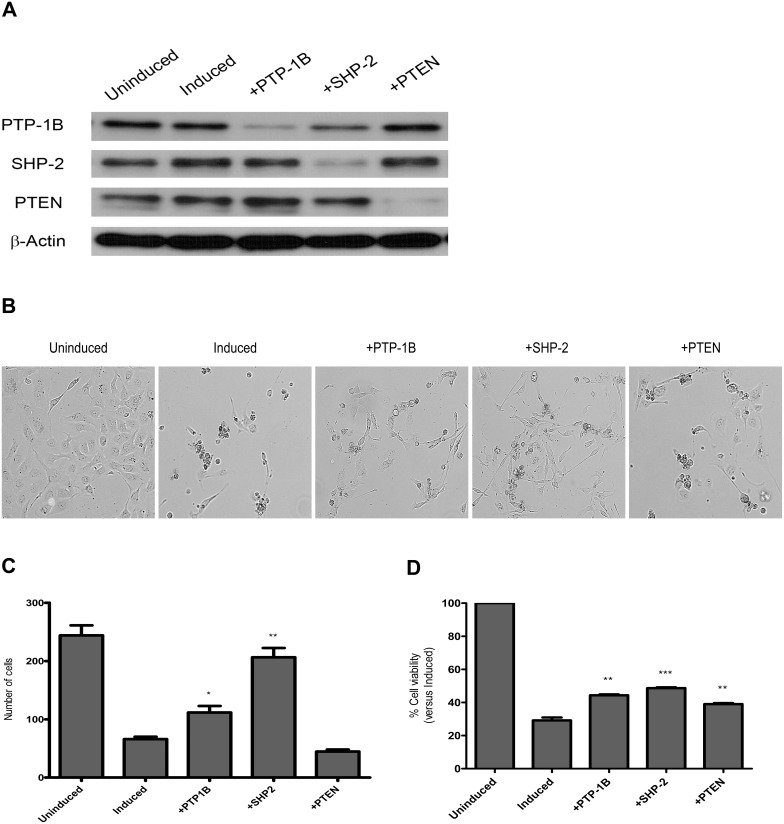
Loss of PTP expression in apoptotic cells leads to an increase in cell proliferation. PTP gene expression was silenced in ER-dnE2F1 cells with siRNA specific for PTP-1B (+PTP-1B), SHP-2 (+SHP-2), or PTEN (+PTEN). Uninduced and induced control cells were transfected with scrambled siRNA. Then cells were treated with 4-OHT for 48 h to initiate Rb/E2F-associated apoptosis. Uninduced control cells were left untreated. (A) Total protein was isolated from lysated cells, resolved by SDS-PAGE, and immunoblotted with antibodies specific for PTP-1B, SHP-2, PTEN and β-actin. (B) Morphological changes in uninduced and induced ER-dnE2F1 cells were compared with morphological changes in induced cells after knockdown of PTPs. (C) The number of cells within three random fields (100X) were counted and results are expressed as the mean + standard deviation. (D) Cell viability was determined by WST-8 assay in two independent experiments performed in triplicate. Results are expressed as the mean + standard deviation. (*) *p*<0.05; (**) *p*<0.01; (***) *p*<0.001.

In order to characterize the function of PTP-1B, SHP-2 or PTEN in Rb/E2F-associated apoptosis, we utilized gene-specific small interfering RNA (siRNA) to silence expression of the three PTPs in ER-dnE2F1 cells. Western blot analysis confirmed that transfection of the ER-dnE2F1 cells with siRNA reduced protein expression of PTP-1B, SHP-2, and PTEN for 48 h after induction of Rb/E2F-associated apoptosis (Fig. 3A). Based on morphological assessments, the knockdown of PTP-1B, SHP-2, or PTEN expression in induced ER-dnE2F1 cells had an effect on cell survival as shown in the representative images (Fig. 3B). The average number of cells per random field of view (n = 3) significantly increased in induced cells transfected with PTP-1B siRNA (*p* = 0.04) or SHP-2 siRNA (*p*<0.01) in comparison to induced cells transfected with scrambled (control) siRNA (Fig. 3C). For PTP-1B, the average number of cells increased from 66 cells in induced to 112 cells in induced with PTP-1B siRNA and for SHP-2, the average number of cells increased to 206 cells, which is comparable to the average number of uninduced control cells. For PTEN, a significant change in average cell number was not visible. Quantification of actual cell viability of ER-dnE2F1 cells undergoing Rb/E2F-associated apoptosis after treatment with PTP-specific siRNA was performed using colorimetric assays which directly measure cellular metabolic activity. The data showed that cell viability of induced cells significantly increased with knockdown of PTP-1B (*p*<0.01), SHP-2 (*p*<0.001), or PTEN (*p*<0.01) expression relative to control (Fig. 3D). The greatest effect was seen with the loss of SHP-2: cell viability increased from 29% for induced cells transfected with scrambled siRNA to 49% for induced cells transfected with SHP-2 siRNA. Induced cells treated with PTP-1B siRNA showed an increase in cell viability from 29% to 44% while induced cells treated with PTEN siRNA showed an increase from 29% to 39%. No change in cell viability was seen in uninduced ER-dnE2F1 cells transfected with PTP-specific siRNA (Data not shown). The results demonstrate that loss of PTP-1B, SHP-2, or PTEN expression during Rb/E2F-associated apoptosis allows cells to survive the apoptotic response, implying that the direct activation of these three PTPs following the loss of Rb/E2F repression may be important for the regulation of the Rb/E2F-associated apoptotic pathway.

### Activation of PTP-1B, SHP-2, and PTEN Promotes Caspase Cleavage

**Figure 4:**
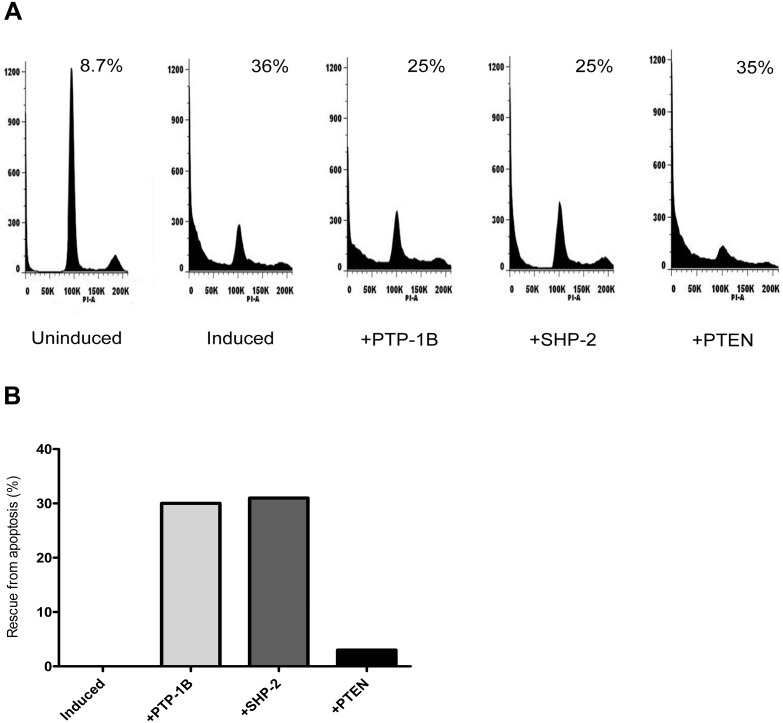
Knockdown of PTP expression rescues cells from Rb/E2F-associated apoptosis. ER-dnE2F1 cells were transfected with siRNA specific for PTP-1B (+PTP-1B), SHP-2 (+SHP-2), or PTEN (+PTEN). Uninduced and induced control cells were transfected with scrambled siRNA. Then cells were treated with 4-OHT for 48 h to trigger apoptosis. Uninduced control cells were left untreated. (A) DNA content was measured by flow cytometry. (B) Apoptosis rescue is expressed as the reduction of cells with<2N DNA content compared with induced control cells.

To verify whether or not PTP-1B, SHP-2, or PTEN activation is required to facilitate Rb/E2F-associated apoptotic signaling, flow cytometry was performed on uninduced ER-dnE2F1 cells transfected with scrambled siRNA (negative control) and induced cells transfected with either scrambled siRNA (positive control) or PTP-specific siRNA to evaluate the cells for apoptosis. As expected, the data showed that induction of Rb/E2F-associated apoptosis for 48 h lead to an increase in the percentage of cells with a<2N DNA content; the percentage of cells increased from 8.7% for uninduced cells to 36% for induced cells (Fig. 4A). However, the apoptotic response was reversed once expression of PTP-1B, SHP-2, or PTEN was silenced by siRNA in induced cells. In comparison to the positive control, knockdown of PTP-1B rescued induced cells from apoptosis by 29% while knockdown of SHP-2 rescued cells from apoptosis by 31% (Fig. 4B). Knockdown of PTEN had a smaller effect and apoptosis was rescued by only 3%. It is possible that knockdown of PTEN had a greater effect on cell viability than cell death because loss of PTEN would free the PI3K/AKT signaling pathway to promote cell proliferation. Previous data showed that treatment of ER-dnE2F1 cells induced to undergo apoptosis with a pan phosphatase inhibitor completely rescued cells from cell death [23], therefore the correlation of the flow cytometry data on induced cells treated with individual siRNA confirms that PTP-1B, SHP-2, and PTEN play a functional role in Rb/E2F-associated apoptosis.

**Figure 5:**
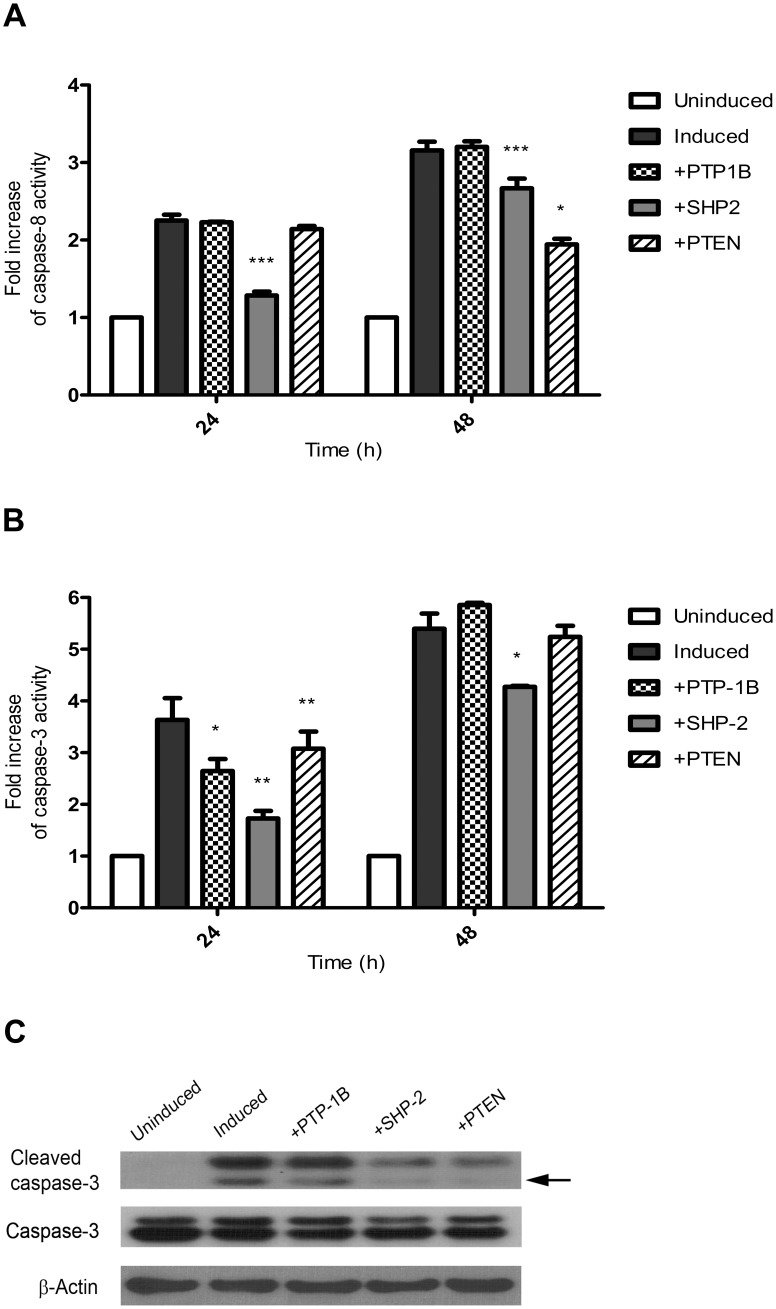
Knockdown of PTP expression leads to a reduction in caspase activity. ER-dnE2F1 cells were transfected with siRNA specific for PTP-1B (+PTP-1B), SHP-2 (+SHP-2), or PTEN (+PTEN). Uninduced and induced control cells were transfected with scrambled siRNA. Then cells were treated with 4-OHT for 24 or 48 h to initiate Rb/E2F-associated apoptosis. Uninduced control cells were left untreated. (A) Caspase-3/CPP32 cleavage was measured by colorimetric assay in three independent experiments. Results are expressed as the mean + standard error. (B) FLICE/Caspase-8 cleavage was measured by colorimetric assay in three independent experiments. Results are expressed as the mean + standard error. (C) Total protein was isolated from lysated cells at 24 h following induction of apoptosis, resolved by SDS-PAGE, and immunoblotted with antibodies specific for cleaved caspase-3 (Denoted by arrow), caspase-3 and β-actin. (*) *p*<0.05; (**) *p* = 0.01; (***) *p*<0.01.

Given that Rb/E2F-associated apoptosis is mediated by caspase-8, ER-dnE2F1 cells also were evaluated for apoptosis by colorimetric assays that directly measure cleavage (i.e. activation) of pro-caspase-8 and pro-caspase-3, a downstream substrate of the initiator caspase caspase-8. At 24 and 48 h, cleavage of caspase-8 in ER-dnE2F1 cells induced to undergo Rb/E2F-associated apoptosis increased 2-fold and 3-fold, respectively, in comparison to uninduced ER-dnE2F1 cells. However, knockdown of SHP-2 with siRNA prior to induction of apoptosis significantly (*p*<0.01) reduced cleavage of caspase-8 in comparison to the induced control at both time points (Fig. 5A). Knockdown of PTEN also reduced cleavage of caspase-8 (*p*<0.05) 48 h after induction of apoptosis. There was no significant change in caspase-8 cleavage with knockdown of PTP-1B. For caspase-3, cleavage increased 4-fold and 5-fold at 24 and 48 h, respectively, between induced and uninduced cells as expected (Fig. 5B). At 24 h, cleavage of caspase- 3 was significantly inhibited by loss of PTP-1B (*p*<0.02), SHP-2 (*p* = 0.01), or PTEN (*p* = 0.01) expression, while at 48 h only SHP-2 had a significant (*p*<0.05) effect on caspase-3 activation. Western blot analysis of cleaved caspase-3 expression after induction of apoptosis in uninduced cells, induced cells, and induced cells transfected with PTP-specific siRNA confirmed the results from the caspase-3 activity assay (Fig. 5C). The results imply that activation of PTP-1B, SHP-2, and PTEN following loss of Rb/E2F repression facilitates the Rb/E2F-associated apoptotic response by promoting caspase activation for the execution of cell death.

## Discussion

The Rb/E2F pathway is essential as it tightly links cell cycle regulation/cell proliferation to apoptosis. The Rb/E2F complex prevents the transcription of several pro-proliferative and pro-apoptotic genes. Mutation(s) that result in loss-of-function of Rb can lead to hyperactivation of these genes and, subsequently, to cellular immortalization and transformation. Tumors containing loss-of-function mutations for Rb often have additional genetic alterations that allow cells to evade apoptosis [10]. The inactivation of the apoptotic response plays a critical role in carcinogenesis. Many cancers possess inactivating mutations for pro-apoptotic proteins [3], [6]. Cancers that are capable of evading apoptosis are more difficult to treat because they are often resistant to chemotherapeutic agents which work by triggering cell death [6]. E2F1 mediated apoptosis can act as an important fail-safe mechanism for the elimination of hyperproliferative cells and for the suppression of cellular transformation and tumor formation following disruption of Rb function.

Our investigation further characterizes the Rb/E2F-associated apoptotic signaling pathway, a p53-independent mechanism. Previously, we had shown that transcriptional expression of *PTPN1* (PTP-1B) and *PTPN11* (SHP-2) was de-repressed in association with Rb/E2F-associated apoptosis, and this finding provided evidence that PTPs potentially play an important role in this apoptotic pathway [23], [32]. Our current data demonstrates that Rb/E2F de-repression is sufficient to also promote expression of *PTEN* tumor suppressor. Furthermore, we demonstrate that PTP-1B, SHP-2, and PTEN play a functional role in Rb/E2F-associated apoptosis by promoting caspase activation, implying that PTP-1B, SHP-2 and PTEN can play a role in tumor suppression through their function in this apoptotic pathway.

In recent years protein tyrosine phosphatases have been intensely investigated to determine their tumor suppressive capabilities given that they negatively regulate protein tyrosine kinase activity, a major contributor to human cancers [1], [32], [44]–[49]. PTP-1B has been shown by several groups to function as either a proto-oncogene or a tumor suppressor depending on its substrate which varies based on cell type and context; its expression is increased in human breast, ovarian and epithelial carcinomas, and it is decreased in oesophageal carcinomas [47], [50]–[52]. The role of PTP-1B as a tumor suppressor is supported by our data which shows that PTP-1B expression is increased following activation of Rb/E2F-associated apoptosis. By specifically silencing PTP-1B we were able to observe an increase in cell viability and a significant decrease in cell death (Fig. 3 & 4). siRNA knock down of PTP-1B also resulted in decreased activity of the apoptotic executioner caspase-3 (Fig. 5).

The phosphatase SHP-2 has been characterized as an oncogene [46], [53], [54]; however, new studies have shown that SHP-2 may act as a tumor suppressor in hepatocellular carcinoma (HCC): deletion of SHP-2 in hepatocytes led to an increase in the development of hepatocarcinoma *in vivo* and *in vitro*; in agreement with this data a subpopulation of human HCC samples showed decreased SHP-2 expression [55]–[57]. Our results also suggest SHP-2 may act as a tumor suppressor through its function in Rb/E2F-associated apoptosis. Initiation of Rb/E2F-associated apoptosis yields a significant increase in SHP-2 expression, suggesting SHP-2 plays a physiological role in this apoptotic response (Fig. 2). Loss of SHP-2 expression leads to a significant increase in cell proliferation and a significant decrease in apoptosis (Fig. 3 & 4). The decrease in apoptosis coincided with a significant decrease in both caspase-8 and caspase-3 activity (Fig. 5). This data indicates that SHP-2 is required to facilitate the Rb/E2F-associated apoptotic response. As in many cancers, Rb function is disrupted in most HCC [57], [58]. Recent studies have demonstrated that FAK is overexpressed in HCC and can play a role in tumor progression and invasion [24], [25], [31]. It is possible that a mechanism similar to the one we describe may contribute to heptatocellular carcinogenesis. We believe the concurrent de-regulation of Rb and SHP-2 could promote tumorigenesis through the failure of the Rb/E2F-associated apoptotic response to eliminate hyperproliferative transformed cells.

Perhaps the most intriguing results we obtained from our investigation involved PTEN. PTEN is a well-known and widely studied tumor suppressor. It has been strongly associated with maintaining genomic stability, and studies have shown that *PTEN*-deficient cells demonstrate increased proliferation, reduced apoptosis, and increased migration [33], [40]. It is assumed that PTEN functions as a tumor suppressor primarily through its negative regulation of the PI3K/AKT cell survival signaling pathway [33], [34], [37], [39], [59]. Our findings suggest that PTEN can possess a novel tumor suppressive function in an Rb/E2F-associated signaling pathway. For the first time, we showed that E2F1 specifically binds to the *PTEN* promoter so loss of Rb/E2F transcriptional repression led to expression of *PTEN*, which correlated with an increase in PTEN protein expression (Fig. 1 & 2). Specifically knocking down PTEN significantly increased cell proliferation and decreased apoptosis (Fig. 3 & 4) and it significantly decreased caspase-8 activity and caspase-3 activity following induction of Rb/E2F-associated apoptosis (Fig. 5). Together, these data suggest that PTEN can function in an Rb/E2F-associated apoptotic pathway to eliminate hyperproliferative cells. Interestingly, qPCR analysis and western blot analysis showed that PTEN protein expression further increased 24 h after induction of Rb/E2F-associated apoptosis, suggesting that PTEN also may be further regulated by downstream proteins that are either activated or inactivated during the progression of the apoptotic response and providing additional evidence that PTEN plays an important role in this pathway.

**Figure 6:**
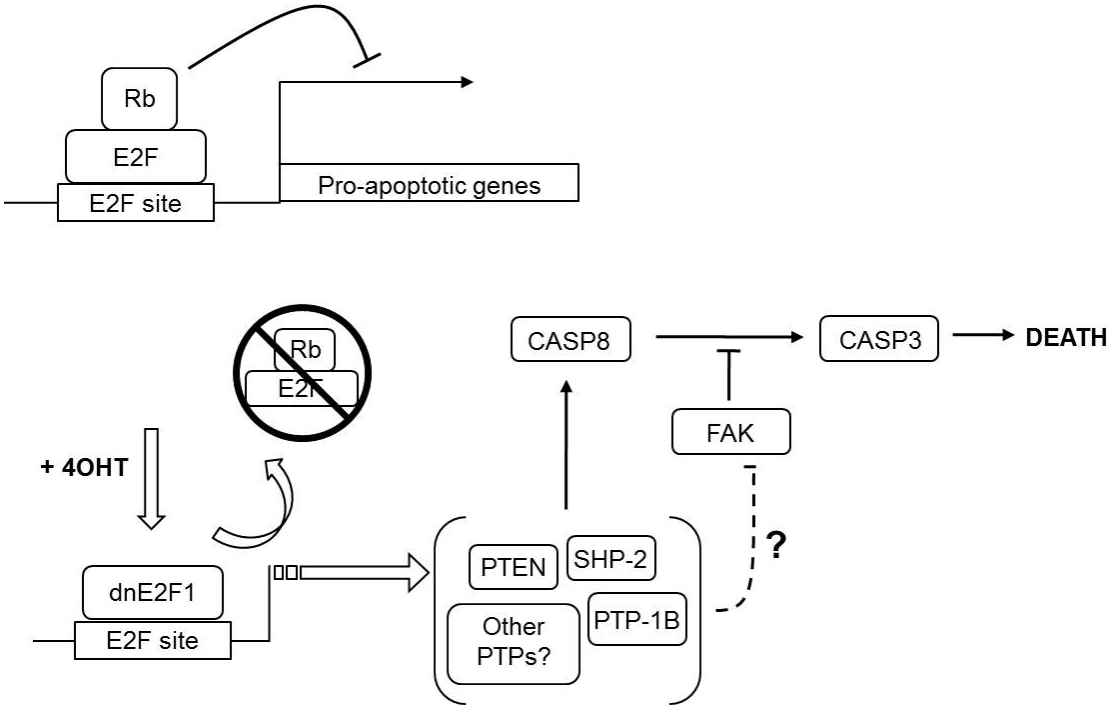
Overview of the Rb/E2F-associated apoptotic pathway. Active Rb/E2F complexes bind to E2F sites within the promoters of several pro-apoptotic genes, repressing their transcription. Treatment of the U2OS ER-dnE2F1 cell line with 4-OHT results in the translocation of dnE2F1 to the nucleus where it displaces Rb/E2F complexes from their binding sites. The loss of Rb/E2F repression leads to the up-regulation of PTP-1B, SHP-2, and PTEN (and potentially other phosphatases), dephosphorylation of FAK, and stimulation of apoptosis through activation of caspase-8.

In our system, p16Ink4a is activated in an inducible cell line by treatment with IPTG, leading to activation of Rb and the formation of the Rb/E2F complex. Formation of the Rb/E2F complex inhibits transactivation of both pro-proliferative genes and pro-apoptotic genes like p19_ARF_ (for the p53 pathway) and it represses transcription of other E2F-responsive genes. Addition of tamoxifen induces overexpression of dnE2F1 which can displace the Rb/E2F complex to simulate loss of Rb/E2F transcriptional repression without stimulating the pathway(s) involving transactivation. Loss of Rb/E2F repression leads to the dephosphorylation of FAK and the subsequent Rb/E2F-associated apoptotic response [23]. Our data suggest a model in which PTP-1B, SHP-2, and PTEN are normally repressed by Rb/E2F complexes (Fig. 6). In the event Rb function becomes deregulated, these PTPs can be activated as part of the Rb/E2F-associated apoptotic signaling pathway, an important mechanism that all cancers must overcome for cellular transformation and tumorigenesis. Since PTP-1B, SHP-2 and PTEN have been implicated in the regulation of FAK [23], [33]–[35], [53], [54], [60], [61], it can be inferred from the results of that these three phosphatases may promote caspase activation by inactivating FAK. Our results imply that PTP-1B, SHP-2, and PTEN may co-operate with one another (and most likely other PTPs) to facilitate the Rb/E2F-associated apoptotic response. Identifying the proteins involved in this pathway contributes to the identification of potential novel targets for the development of more specific and effective anti-cancer drugs and therapies.
